# Assessing the criteria for definition of perimembranous ventricular septal defects in light of the search for consensus

**DOI:** 10.1186/s13023-019-1044-2

**Published:** 2019-04-03

**Authors:** Justin T. Tretter, Vi-Hue Tran, Seth Gray, Hieu Ta, Rohit S. Loomba, William O’Connor, Diane E. Spicer, Andrew C. Cook, Robert H. Anderson

**Affiliations:** 1 The Heart Institute, Cincinnati Children’s Hospital Medical Center, 3333 Burnet Avenue, Cincinnati, OH 45229 USA; 2 Department of Pediatrics, University of Cincinnati College of Medicine, Cincinnati, OH USA; 3 Institute of Cardiovascular Science, University College London, London, UK; 4 Advocate Children’s Heart Institute, Advocate Children’s Hospital, Oak Lawn, IL USA; 5 Department of Pathology, University of Kentucky Medical Center, Lexington, KY USA; 6 Department of Pediatric Cardiology, University of Florida, Gainesville, Florida, USA; 7 Institute of Genetics, Newcastle University, Newcastle upon Tyne, UK

**Keywords:** Classification, Congenital heart disease, Nomenclature, Perimembranous ventricular septal defect, Ventricular septal defect

## Abstract

**Background:**

Discussions continue as to whether ventricular septal defects are best categorized according to their right ventricular geography or their borders. This is especially true when considering the perimembranous defect. Our aim, therefore, was to establish the phenotypic feature of the perimembranous defect, and to establish the ease of distinguishing its geographical variants.

**Methods and results:**

We assessed unrepaired isolated perimembranous ventricular defects from six historic archives, subcategorizing them using the ICD-11 coding system. We identified 365 defects, of which 94 (26%) were deemed to open centrally, 168 (46%) to open to the outlet, and 84 (23%) to the inlet of the right ventricle, with 19 (5%) being confluent. In all hearts, the unifying phenotypic feature was fibrous continuity between the leaflets of the mitral and tricuspid valves. This was often directly between the valves, but in all instances incorporated continuity through the atrioventricular portion of the membranous septum. In contrast, we observed fibrous continuity between the leaflets of the tricuspid and aortic valves in only 298 (82%) of the specimens. When found, discontinuity most commonly was seen in the outlet and central defects. There were no discrepancies between evaluators in distinguishing the borders, but there was occasional disagreement in determining the right ventricular geography of the defect.

**Conclusions:**

The unifying feature of perimembranous defects, rather than being aortic-to-tricuspid valvar fibrous continuity, is fibrous continuity between the leaflets of the atrioventricular valves. While right ventricular geography is important in classification, it is the borders which are more objectively defined.

## Introduction

Excluding the aortic valve with two leaflets, isolated ventricular septal defects constitute the commonest form of congenital cardiac disease [1]. Despite their frequency, their description has become a tower of Babel. Various systems of nomenclature are in use, often within the same institution, with many of the systems being based on expert opinion rather than evidence. The International Society for Nomenclature of Paediatric and Congenital Heart Disease (ISNPCHD) was formed in 2000 with the goal of formulating a universally acceptable, and evidence-based, system of nomenclature for congenital heart disease. Their efforts have led to the acceptance of their proposed system by the World Health Organization for the 11th Iteration of the International Classification of Diseases (ICD-11) [2]. Using this classification, the phenotypic variants of ventricular septal defects are categorized both on the basis of their right ventricular geography and the borders of the defect. Further sub-classification is then based on the presence or absence of septal malalignment. Although there is general agreement regarding the definitions to be used for the muscular and doubly committed and juxta-arterial variants, the perimembranous defect continues to pose the main challenge in classification [2, 3]. This latter defect is the most frequent of the three major phenotypic variants as categorized on the basis of their borders. With this in mind, we have analyzed in systematic fashion the specimens with this phenotype collected in multiple centers in the setting of concordant atrioventricular and ventriculo-arterial connections. Our aim was to establish the uniform feature permitting their recognition, and to establish the ease of distinguishing the geographical variants.

## Methods

We systematically analyzed all specimens held in 6 archives, namely Cincinnati Children’s Hospital Medical Center in Cincinnati, Ohio; the Institute of Cardiovascular Sciences, University College, London; the Van Mierop Archive at the University of Florida in Gainesville, Florida; Johns Hopkins All Children’s Hospital in St. Petersburg, Florida; University of Kentucky in Lexington, Kentucky; and the Farouk S. Idriss Registry at the Lurie Children’s Hospital in Chicago, Illinois. We assessed only unrepaired specimens catalogued as having ventricular septal defects in the setting of concordant atrioventricular and ventriculo-arterial connections. Each specimen was analyzed by at least two experienced cardiac anatomists. In instances of discrepancy in assessment, the heart was photographed and analyzed by the group for consensus. We identified the defects deemed to be perimembranous on the basis of the presence of a fibrous postero-inferior border to the defect [4]. We excluded all defects from hearts with other than concordant atrioventricular and ventriculo-arterial connections, along with those that were part of more complex forms of congenital heart disease, such as those with tetralogy of Fallot. We also excluded those that extended also to become doubly committed and juxtaarterial despite the presence postero-inferiorly of a fibrous border. We then sought to sub-classify the defects, as far as was possible, on the basis of the definitions provided for the ICD-11 classification system, specifically:

- The perimembranous central defect occupies the space usually closed by the interventricular part of the membranous septum adjacent to the area of fibrous continuity between the leaflets of the atrioventricular valve and an arterial valve, located at the center of the base of the ventricular mass.
- The perimembranous inlet defect extends from the area of the membranous septum, posteriorly and inferiorly under the septal leaflet of the tricuspid valve. This type of perimembranous defect commonly has alignment between the atrial and muscular ventricular septum, but rarely can have malalignment between these structures with tricuspid valve override with or without straddling.
- The perimembranous outlet defect extends from the area of the membranous septum, anteriorly and superiorly between or above the limbs of the septomarginal trabeculation. The muscular outlet septum can be malaligned in either an antero-cranial or postero-caudal fashion with respect to the trabecular muscular septum.

When finding defects that, on the basis of these definitions, showed both inlet and outlet extension, we categorized them as being confluent, although that definition, as yet, is not to be found in the classification accepted for ICD-11.

Additional features were recorded as follows:

- The size of the defect was qualitatively assessed as compared to the size of the estimated diameter of the virtual basal ring of the aortic root. We categorized the defect as small when the dimension was less than one third the diameter, medium for dimensions between one third and one half, and large when greater than one half.
- In addition to fibrous continuity between the aortic and tricuspid valves, this being the historical defining phenotypic feature of the perimembranous defect as accepted for ICD-11, we recorded the presence of additional direct fibrous continuity between the leaflets of the mitral and tricuspid valves.
- We then assessed carefully the nature of fibrous continuity in the postero-inferior margin of the defect. This was because, in a significant number of hearts, we found indirect fibrous continuity between the leaflets of the tricuspid and mitral valves through the substance of the central fibrous body in the absence of direct continuity between the leaflets of the tricuspid and aortic valves.
- We established the location of the medial papillary muscle, also known as the papillary muscle of the conus or muscle of Lanscisi, when present, relative to the margins of the defect.
- We took particular note of the position of the supraventricular crest relative to the limbs of the septomarginal trabeculation, or septal band.
- We noted the presence or absence of malalignment between the atrial and muscular ventricular septal components, and of the muscular outlet septum relative to the crest of the muscular ventricular septum.

The study was performed in accordance with the World Medical Association Declaration of Helsinki in that consent was obtained for the collection and analysis of human tissue in all involved archives.

## Results

**Fig. 1:**
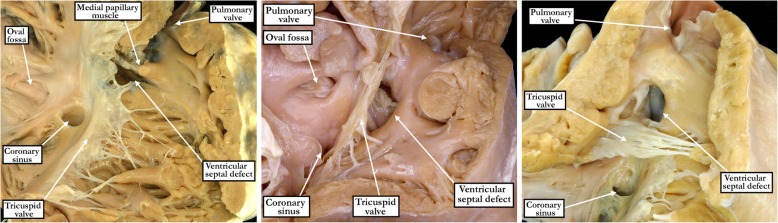
The images show the features of perimembranous defects opening centrally to the base of the right ventricle (left hand panel), or extending so as to open primarily to the right ventricular inlet (central panel) or the right ventricular outlet (right hand panel). The defects were identified as being perimembranous because of the fibrous nature of their postero-inferior borders (see Fig. 2)

From the various archives, we were able to identify 365 unrepaired defects in hearts with concordant atrioventricular and ventriculo-arterial connections in the absence of more complex forms of congenital heart disease (Table 1). Of these, we deemed 94 (26%) to be central (Figs. 1- left hand panel and 2 - left hand panel), 168 (46%) to extend so as to open to the outlet of the right ventricle (Figs. 1- right hand panel and 2 - right hand panel), 84 (23%) to extend to open to the right ventricular inlet (Figs. 1 - central panel and 2 - central panel), and 19 (5%) to be confluent (Fig. 3). Of those defects opening centrally, four-fifths were predominately small (80%), while over nine-tenths of the outlet, inlet and confluent defects were predominately medium to large (94%).

**Table 1:** Perimembranous ventricular septal defect variant characteristics

	Perimembranous (*n* = 365)			
	Central (*n* = 94)	Inlet (*n* = 84)	Outlet (*n* = 168)	Confluent (*n* = 19)
Size	80% small (75), 17% moderate (16), 3% large (3)	13% small (11), 31% moderate (26), 56% large (47)	3% small (5), 29% moderate (49), 68% large (114)	32% moderate (6), 68% large (13)
Tricuspid to Aortic Valve Fibrous Continuity	80% (75)	95% (80)	74% (125)	95% (18)
- Tricuspid valve leaflet involved	55% septal (41), 24% antero-superior and septal (18), 21% antero-superior (16)	92% septal (74), 4% antero-superior and septal (3), 4% anterior (3)	26% septal (32), 10% antero-superior and septal (13), 64% antero-superior (80)	17% septal (3), 61% antero-superior and septal (11), 22% antero-superior (4)
Tricuspid to Mitral Valve Fibrous Continuity	100% (94)	100% (84)	100% (168)	100% (19)

**Fig. 2:**
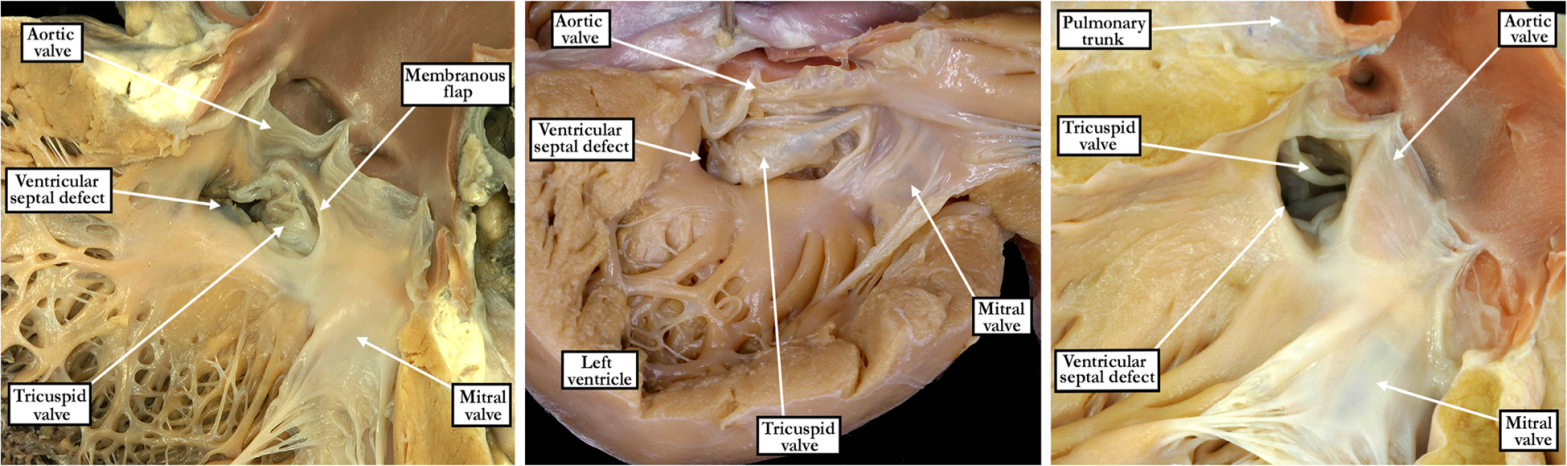
The images show the features of the defects shown in Fig. 1 as viewed from the left ventricle. In each heart, there is a fibrous postero-inferior border. This proved to be the defining feature of the perimembranous defect. In these hearts, the fibrous border is formed of continuity between the leaflets of the aortic, mitral, and tricuspid valves, incorporating also the atrioventricular component of the membranous septum. In the central defect, shown in the left hand panel, there is also a remnant of the interventricular component of the membranous septum, forming the so-called membranous flap. In the central and inlet defects (left hand and central panels), the fibrous continuity involves the septal leaflet of the tricuspud valve, whereas in the outlet defect (right hand panel), there is also continuity with the antero-superior leaflet of the valve in the roof of the defect. The postero-inferior border, nonetheless, is still formed by continuity between the septal leaflet of the tricuspid valve and the mitral valve via the substance of the atrioventricular component of the membranous septum

**Fig. 3:**
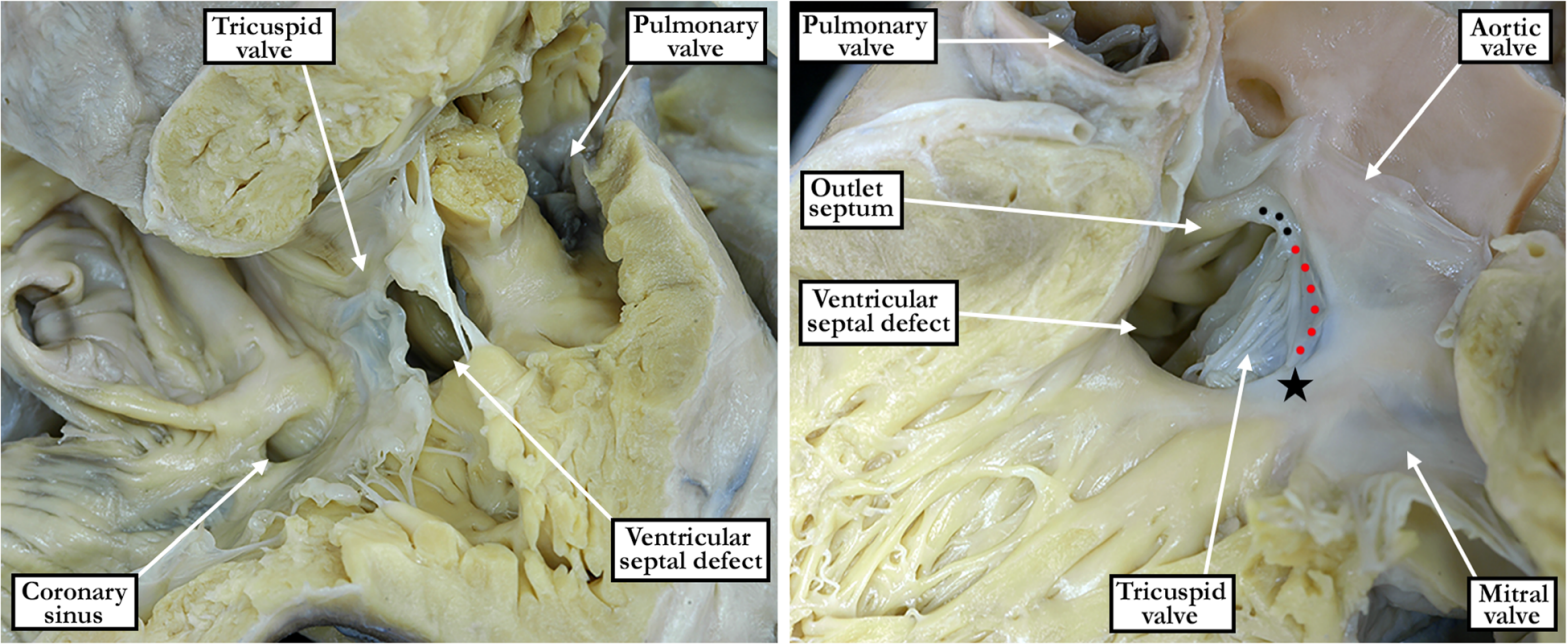
The images show a confluent perimembranous defect as seen from the right ventricle (left hand panel) and the left ventricle (right hand panel). As seen in the right hand panel, there is malalignment of the muscular outlet septum, but the defect also opens to the inlet of the right ventricle. The right hand panel shows the extensive fibrous continuity posteriorly and inferiorly between the leaflets of the aortic, tricuspid and mitral valves (red dotted line), incorporating the atrioventricular component of the membranous septum (black star), but discontinuity cranially between the leaflets of the aortic and tricuspid valves produced by the malaligned muscular outlet septum. Note also the remnant of the interventricular component of the membranous septum in the corner of the roof of the defect (black dotted line)

**Fig. 4:**
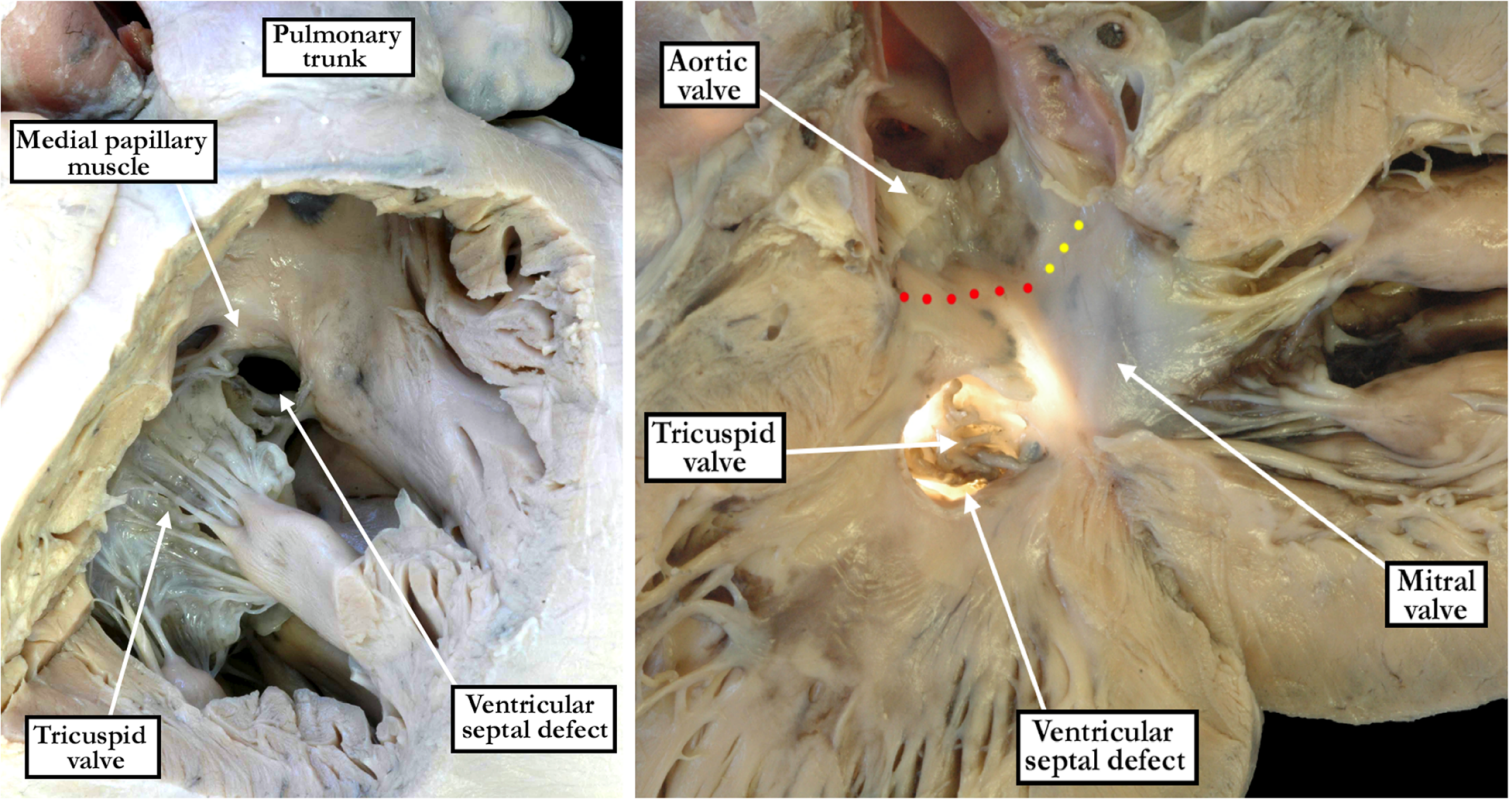
The image in the left hand panel shows a defect opening centrally at the base of the right ventricle, with the supraventricular crest normally inserted between the limbs of the septomarginal trabeculation. The right hand panel shows the left ventricular aspect of the defect. It has been transilluminated to show the atrioventricular component of the membranous septum, which produces fibrous continuity between the leaflets of the tricuspid and mitral valves. There is also fibrous continuity between the leaflets of the aortic and mitral valves (yellow dotted line). Fibrous continuity is lacking, however, between the leaflets of the aortic and tricuspid valves (red dotted line). The defect remains perimembranous because of the tricuspid-to-mitral valvar continuity via the substance of the atrioventricular component of the membranous septum

**Fig. 5:**
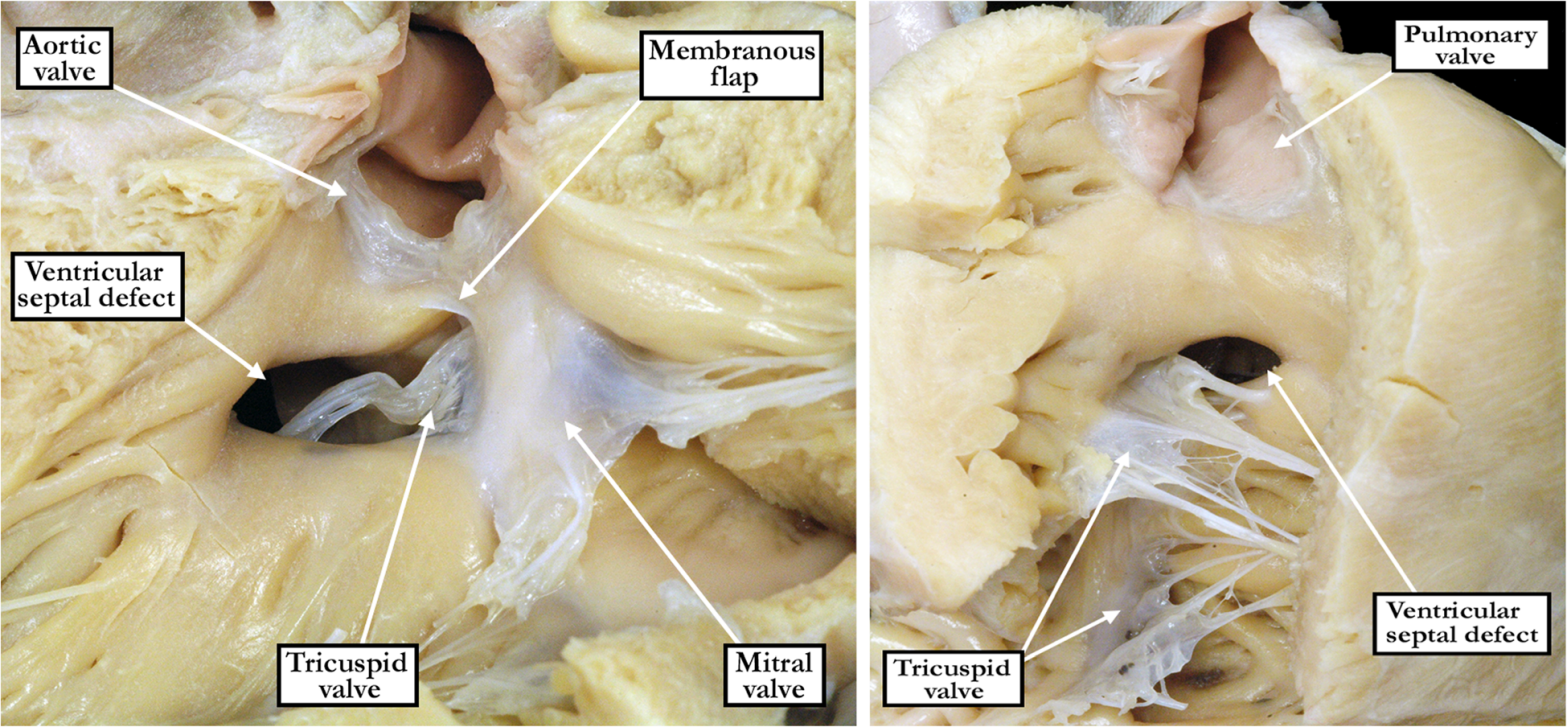
The left hand panel shows another defect viewed from the left ventricle with fibrous continuity postero-inferiorly between the leaflets of the mitral and tricuspid valves, incorporating the atrioventricular component of the membranous septum along with a remnant of the interventricular component forming a membranous flap. The muscular outlet septum, however, which is deviated caudally into the subaortic outflow tract, interposes between the leaflets of the aortic and tricuspid valves. When viewed from the right ventricle (right hand panel), it is difficult to appreciate that the defect opens to the right ventricular outlet, being cephalad relative to the medial papillary muscle

In all specimens, we were able to identify a fibrous postero-inferior border, or floor, to the defects. This fibrous continuity was directly between the leaflets of the tricuspid and mitral valves in all of the perimembranous inlet and confluent defects (Figs. 2 - central panel and 3 - right hand panel). Such direct fibrous continuity between the leaflets of the atrioventricular valves was also found in 91% of the defects opening centrally, and in 53% of those opening to the right ventricular outlet. In those without direct continuity, additional indirect fibrous continuity was found through the part of the central fibrous body formed by the intact atrioventricular portion of the membranous septum and the right fibrous trigone in 9% of the defects opening centrally, and in 47% of the defects opening to the right ventricular outlet (Fig. 2 - right hand panel). Fibrous continuity between the leaflets of the tricuspid and aortic valves, however, was observed in only 298 (82%) of the specimens. Thus, in 19 (20%) of the hearts opening centrally, fibrous continuity was lacking between the leaflets of the aortic and tricuspid valves (Fig. 4). Fibrous continuity between these leaflets was also lacking in 43 (26%) of the defects opening to the right ventricular outlet (Fig. 5 - left hand panel), and in 4 (5%) of those opening to the right ventricular inlet. Aortic-to-tricuspid fibrous continuity was also lacking in one of the confluent defects (5%). Of the hearts with outlet extension and discontinuity between the leaflets of the aortic and tricuspid valves, most exhibited postero-caudal malalignment of the outlet septum (33 of 43 specimens, 77% - Fig. 5). In these specimens, it proved difficult, when viewing the hearts from the right ventricle, to appreciate that the defect opened to the outlet component of the right ventricle (Fig. 5 - right hand panel). In those hearts which did have tricuspid-to-aortic fibrous continuity, the continuity with the aortic valve involved predominately the septal leaflet of the tricuspid valve in those deemed central (55% - Fig. 2 - left hand panel), as it did in those opening to the right ventricular inlet (92% - Fig. 2 - central panel). The antero-superior leaflet was mostly involved in those opening to the right ventricular outlet (64% - Fig. 2 - right hand panel). Both the septal and antero-superior leaflets were commonly in continuity with the aortic leaflet in confluent defects (61% - Fig. 3 - right hand panel). There were no discrepancies between the observers in regards to the borders of the defect. The greatest discrepancy was in judging whether there was minimal extension to the inlet or outlet, as opposed to the defect being deemed to open centrally.

**Fig. 6:**
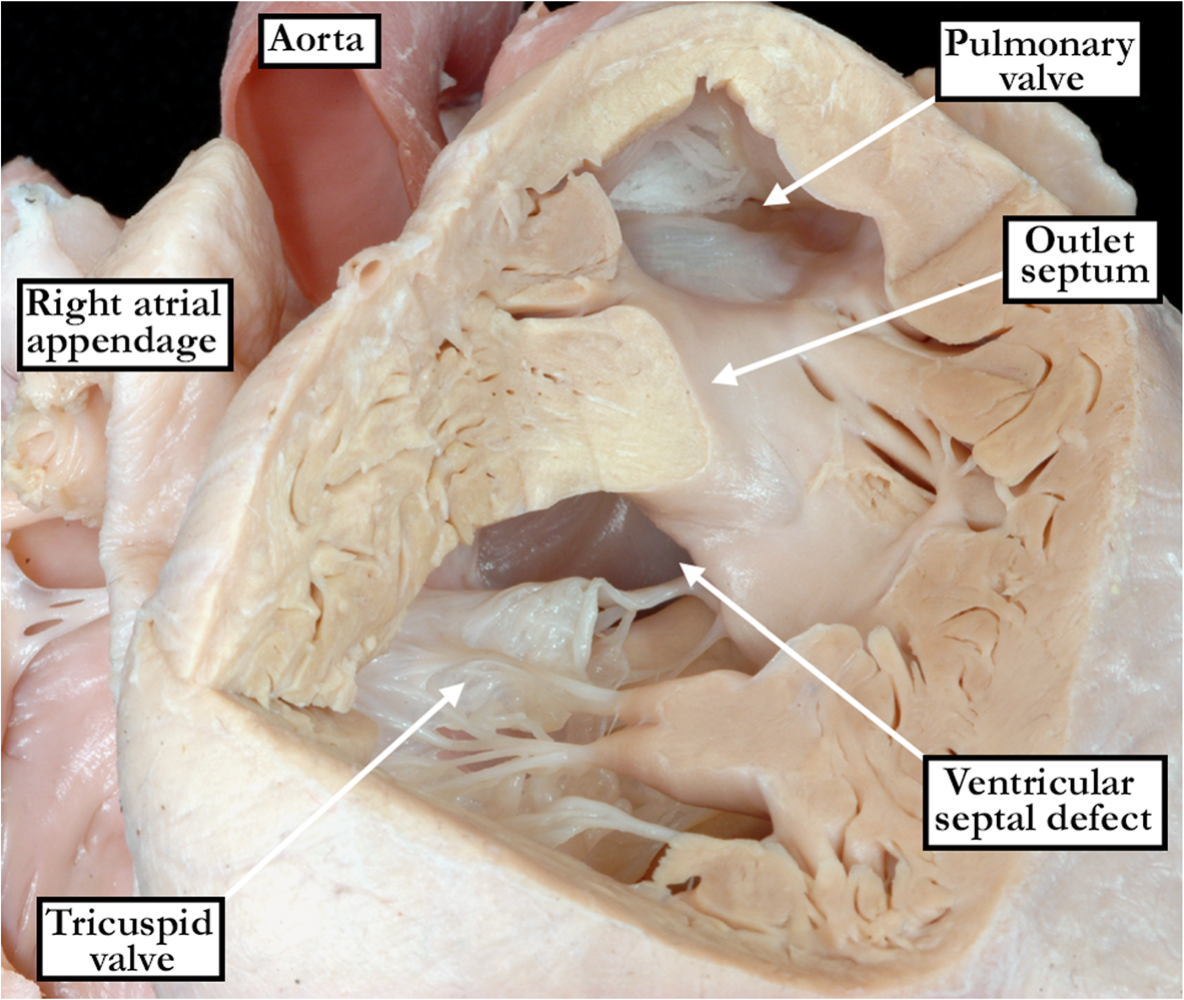
The image shows a perimembranous defect opening to the outlet of the right ventricle with antero-cephalad malalignment of the muscular outlet septum. There is no obstruction, however, of the right ventricular outflow tract. This is an example of the so-called Eisenmenger defect

When the defects opened to the right ventricular outlet, it was usual to find associated malalignment of the muscular outlet septum. This was observed in 156 of the 168 specimens considered to open to the right ventricular outlet (93%). The malalignment was antero-cephaled in 110 specimens (71% - Fig. 6), and postero-caudal in 46 specimens (29% - Fig. 5). At least one additional left-sided lesion, such as an aortic valve with two leaflets, a subaortic fibrous shelf, coarctation of the aorta, or interruption of the aortic arch, was present in 20 of the 46 (43%) specimens with postero-caudal malalignment of the outlet septum. The muscular outlet septum, which was recognizable as forming the margin of the supraventricular crest as part of the border of the defect, was divorced from its usual position within the limbs of the septomarginal trabeculation in the outlet defects with malalignment (Figs. 5 and 6). In those opening to the outlet without malalignment, the supraventricular crest, defined as the myocardial structures extending from the cranial border of the defect to the leaflets of the pulmonary valve, itself was hypoplastic. The crest was malaligned in 12 of the 19 confluent perimembranous defects (63%). Of these, 9 showed malalignment in antero-cephalad direction (75%), and 3 in postero-caudal (25%) direction. All of the latter were associated with left-sided lesions. There was some degree of override of the aortic valve over the crest of the muscular ventricular septum in all specimens with antero-cephalad malalignment of the outlet septum.

**Fig. 7:**
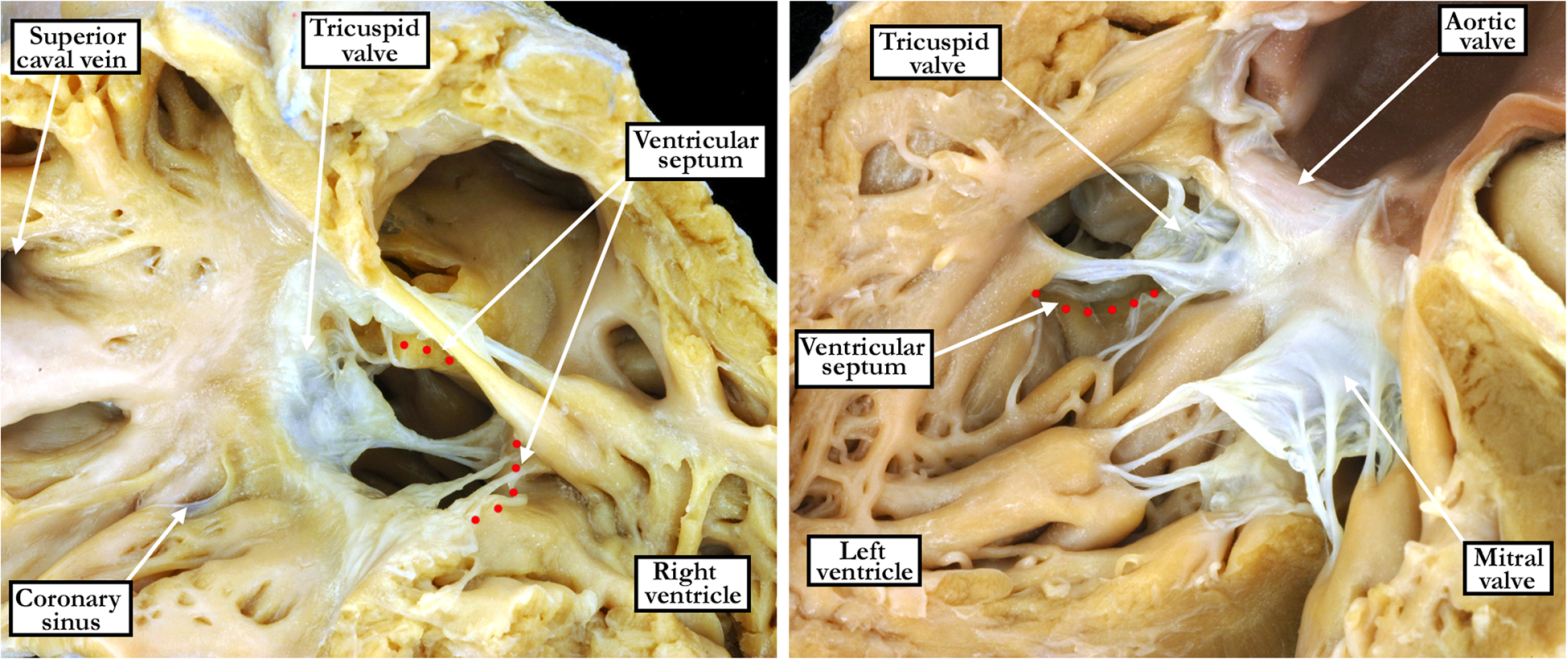
The images show a perimembranous defect opening to the right ventricular inlet in the setting of malalignment between the atrial septum and the crest of the muscular ventricular septum, which is marked by the red dotted lines. The left panel shows the view from the right ventricle, with the right panel showing the left ventricular view. There is straddling and overriding of the tricuspid valve

Malalignment between the atrial and muscular ventricular septal components (Fig. 7) was observed in 5 (6%) of the defects opening to the right ventricular inlet, and in 2 (11%) of the confluent defects. All of these were associated with override and straddling of the tricuspid valve. In all inlet and confluent perimembranous defects, there was also inferior displacement of the apex of the triangle of Koch, apart from those with malalignment of the muscular ventricular septum. In these latter hearts, the triangle of Koch no longer serves as a landmark to the location of the atrioventricular node. The medial papillary muscle, when identifiable, was highly variable in both its size and the presence of single or multiple heads, comparable to the findings seen in the normal heart [5]. Its position relative to the defects themselves was also highly variable. This included whether it attached near the margin of the defect, or was more remote from the margin. In those considered to open to the right ventricular inlet, it commonly resided at either the superior or anterior margin of the defect. In those opening centrally, it could attach at either the inferior, anterior, or superior margin. In those opening to the outlet of the right ventricle, it attached mostly at the inferior margin. In those considered to be confluent it attached mostly at either the inferior or anterior margins.

## Discussion

The document now published by the ISNPCHD has summarized the problems that continue to exist in providing a universally acceptable categorization of hearts with deficient ventricular septation. In that work, it was pointed out that disagreements largely related to whether geography, as opposed to borders, should be paramount when making classifications. If there was to be consensus, it was stressed that both features required description [2]. As was emphasised, only by describing both aspects can the full information be provided so as to achieve optimal diagnosis and treatment for patients with ventricular septal defects. The suggested terms for categorization were grounded on the initial assessment based on geography, with defects grouped according to whether they opened centrally to the right ventricle, or extended to open to the right ventricular inlet or outlet components, recognizing that defects could also open at various sites within the apical muscular septum. The suggested categorizations then moved to discriminating the defects within the various groups according to their borders, noting also any malalignment between the septal components. We designed our current investigation with this proposed system in mind, using hearts stored in multiple historical archives. Our aim was to assess the utility and reproducibility of a system that begins with the description of geography. We reasoned that assessment of the location of a given defect relative to the landmarks of the ventricular mass would be easier, and more accurate, when assessing cadaveric specimens than would be possible in the clinical situation. We purposely limited our approach to investigation of so-called perimembranous defects, since these were the defects identified as continuing to create problems in the search for consensus. We had restricted our choice of specimens for study in this fashion since we further reasoned that defects with exclusively muscular borders would more readily be distinguished during clinical investigation on the basis of their geographical location within the ventricular septum. We also excluded defects of the third type, namely those roofed by fibrous continuity between the leaflets of the aortic and pulmonary valves, since these, of necessity, are outlet defects. In the definitions for the perimembranous defect as proposed in the consensus document, however, with this definition currently accepted for ICD-11, the phenotypic feature has been the presence of fibrous continuity between the leaflets of the tricuspid and aortic valves in their postero-inferior border. As we will discuss further, had we followed this definition we would have been forced to exclude a significant number of hearts having an unequivocal fibrous postero-inferior border.

We were able to collect 365 hearts with fibrous postero-inferior borders from the various archives. All hearts were then examined on the basis of a previously agreed protocol. We were surprised at the difficulties that arose in agreeing on the precise geographical location of the defects present. We were also surprised to find that a significant number of hearts in each of the archives did not exhibit the finding of fibrous continuity between the leaflets of the aortic and tricuspid valves. In all of the hearts examined, nonetheless, we did find fibrous tissue at the postero-inferior border, or floor of the defects. In many of the specimens, this area of fibrous continuity was not directly between the leaflets of the mitral and tricuspid valves. The leaflets were involved in the area, nonetheless, since it included either the atrioventricular component of the membranous septum, or else this part of the membranous septum along with the remnant of its interventricular component. The majority of the hearts, however, did also demonstrate fibrous continuity between the leaflets of the aortic and tricuspid valves. In the significant minority that lacked aortic-to-tricuspid continuity, it was the supraventricular crest, specifically the muscular outlet septum, which interposed between these leaflets. The myocardial component could be identified as the muscular outlet septum when viewed from the left ventricle, since it unequivocally interposed between the ventricular outflow tracts. When assessed from the right ventricle, the myocardial tissues included also a component representing the free-standing muscular infundibular sleeve. Hearts with only mitral-to-tricuspid valvar continuity in the absence of tricuspid-to-aortic continuity were more commonly found when the defects themselves either opened centrally, or opened to the outlet of the right ventricle. In the majority of these hearts, the outlet septal component of the supraventricular crest was deviated caudally. This produced a degree of obstruction of the subaortic outflow tract. The hearts with the greatest degree of obstruction also had either severe aortic coarctation or interruption of the aortic arch. It was not always possible, however, when viewing the hearts from the right ventricular aspect, to identify malalignment of the supraventricular crest relative to the crest of the muscular ventricular septum.

We encountered our greatest degree of difficulty in identifying specific features so as to discriminate between the defects opening centrally, as opposed to those opening either to the outlet or the inlet of the right ventricle. It was an easy matter to identify the defects opening to the outlet of the right ventricle when the supraventricular crest was deviated in antero-cephalad fashion, this being the phenotypic feature of the so-called Eisenmenger defect [6]. These hearts, in the majority of cases, had obvious fibrous continuity between the leaflets of the aortic and tricuspid valves. All, nonetheless, also had fibrous continuity between the leaflets of the mitral and tricuspid valves, albeit on occasion through the substance of the central fibrous body. We had initially thought that the latter feature would permit identification of the perimembranous defects that opened to the inlet of the right ventricle. The finding of such continuity between the leaflets of the atrioventricular valves, either directly or indirectly, in all the perimembranous defects obviously reduced markedly the value of this feature. It was particularly difficult to determine which defects opened centrally, since even those with inlet or outlet extension, when considered in terms of their geography, included a part of the center of the base of the right ventricle as part of their borders. Some of the defects, furthermore, extended so as to open both to the inlet and the outlet of the right ventricle, as well as including the central component. In the light of these caveats regarding the definition of the geographical location of the defects, the most significant finding of our investigation was that, due to the presence of either direct or indirect mitral-to-tricuspid continuity, all of the defects were perimembranous. This constant feature of the perimembranous defect is in line with our recent developmental findings. These show that the tertiary interventricular foramen is bordered by fibrous continuity between the cushions which develop into the leaflets of the atrioventricular valves. Failure of this tertiary interventricular foramen to close underlies the development of the perimembranous defect [7]. Recognition of the fact that a defect is perimembranous, therefore, carries with it the additional crucial information that the atrioventricular conduction axis, which penetrates through the central fibrous body, will be at risk in this postero-inferior border [8].

Simple description of the geography of any given defect, of course, gives no specific information regarding the location of the atrioventricular conduction axis. Defects opening to the inlet of the right ventricle can have the axis positioned postero-inferiorly when they are perimembranous, but antero-superiorly when muscular. In the setting of inlet perimembranous defects with atrioventricular septal malalignment, furthermore, although the conduction axis runs postero-inferiorly, it takes its origin from an anomalous atrioventricular node [9]. Similar problems exist if defects are simply categorized as opening to the outlet of the right ventricle. Outlet defects can be perimembranous, in which case the conduction axis will be directly at risk in their postero-inferior border. Outlet defects, however, can also be muscular or doubly committed. The muscular defects, and the majority of those that are doubly committed, have a myocardial boundary that protects the conduction axis [10]. It might be considered that, by avoiding the postero-inferior border of such defects, the axis would be safe from iatrogenic damage. Perhaps paradoxically, however, deviating the suture line away from an exclusively myocardial border of the defect is more likely to court damaging the right bundle branch as it emerges through the caudal limb of the septal band/septomarginal trabeculation.

## Conclusions

For all of these reasons, therefore, the findings of our investigation suggest that the critical feature of any individual defect in terms of its categorization is the nature of its borders. It is this feature that determines the phenotype, and in turn, carries crucial information regarding the location of the atrioventricular conduction axis [8–10]. We agree with the conclusions of the “consensus document” that it is always necessary to account for both geography and borders of any defect [2]. Our results show, nonetheless, that it is easier, when examining anatomic specimens, to distinguish with precision the borders as opposed to the direction of opening of any defect to the right ventricle. Importantly, our findings also show that the unifying feature of perimembranous defects, rather than being aortic-to-tricuspid valvar fibrous continuity, is fibrous continuity between the leaflets of the mitral and tricuspid valves creating a fibrous floor to the defect. This latter feature, furthermore, holds good for categorization of such defects in hearts with different segmental combinations, such as double outlet right ventricle or transposition [11].

In an ideal world, moreover, it would have proved possible to assess the clinical features of the patients from whom our specimens were obtained. By the very nature of our collection of data, which depends on the availability of historical archives, clinical details such as the genetic background, or the syndromic as opposed to sporadic nature of the cases, are impossible to obtain in retrospective fashion. Such data, nonetheless, can be obtained in future clinical studies. The full relevance of the data, however, will only be achieved if patients are sub-categorized on the basis of the borders of their ventricular septal defects. As we have emphasized, it is only when attention is directed to the borders of the defects as the starting point of segregation, as opposed to their geographical location within the ventricular mass, that it becomes possible to recognize the key phenotypic variations. It is only by recognition of these phenotypic differences that it will prove possible to establish the significance of genetic or syndromic variations.
